# Timing Considerations for Sleeve Gastrectomy in Kidney Transplant Patients: A Single Center Evaluation

**DOI:** 10.3389/ti.2024.12690

**Published:** 2024-06-18

**Authors:** Mario Spaggiari, Alessandro Martinino, Giulia Bencini, Mario A. Masrur, Egor Petrochenkov, Amy Lian, Joanna Olazar, Pierpaolo Di Cocco, Jorge Almario-Alvarez, Enrico Benedetti, Ivo Tzvetanov

**Affiliations:** ^1^ Division of Transplantation, Department of Surgery, University of Illinois at Chicago, Chicago, IL, United States; ^2^ Division of General, Minimally Invasive and Robotic Surgery, Department of Surgery, University of Illinois at Chicago, Chicago, IL, United States; ^3^ University of Illinois at Chicago College of Medicine, Chicago, IL, United States

**Keywords:** kidney transplant, sleeve gastrectomy, timing, weight loss, robotic

## Abstract

Current scientific literature is deficient in detailing the optimal timing for conducting bariatric surgery in relation to kidney transplantation. In this study, we performed a retrospective evaluation of kidney transplant recipients with BMI >35 kg/m^2^. It aimed to provide data on those who received both sleeve gastrectomy (SG) and kidney transplantation (KT) simultaneously, as well as on patients who underwent SG and KT at different times, either before or after. In addition, the acceptance levels of the bariatric surgery among different scenarios were assessed. Our findings demonstrated that combined KT and SG led to successful weight loss, in contrast to undergoing kidney transplant alone, while maintaining comparable rates of graft and patient survival. Weight loss was similar between recipients who had a combined operation and those who underwent SG following the transplant. Additionally, over a median time frame of 1.7 years, patients who underwent SG before KT exhibited a statistically significant reduction in BMI at the time of the transplant. Notably, our study highlights that patients offered the combined procedure were significantly more likely to undergo SG compared to those for whom SG was presented at a different operative time than the transplant.

## Graphical Abstract

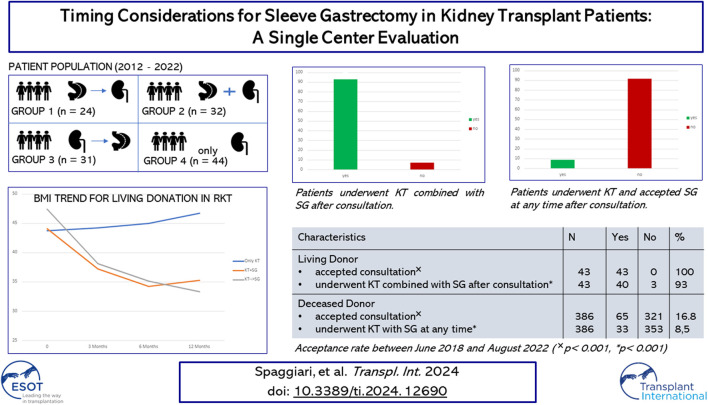

## Introduction

Obesity has emerged as a global epidemic, affecting approximately 13% of the world’s adult population in 2016, a nearly threefold rise over the past four decades [1]. In the last 30 years, bariatric surgery has been established as the paramount therapeutic intervention for weight loss, specifically indicated for class III obesity and for class II obesity when accompanied by a concurrent medical condition [2, 3].

End-stage renal disease is a terminal condition characterized by a glomerular filtration rate of less than 15 mL/min. In the United States, diabetic nephropathy ranks as the most prevalent cause of ESRD, followed by hypertension [4]. Obesity contributes to the onset of non-communicable illnesses such as arterial hypertension (AHT), diabetes mellitus (DM), and atherosclerosis, all factors that also affect the development of CKD, ultimately leading to the progression to end-stage renal disease (ESRD) [5–7]. The effectiveness of kidney transplantation as the primary therapeutic approach for most ESRD patients has been extensively demonstrated, however, with the growing number and complexity of potential recipients, continuous refinement of selection criteria becomes imperative [8–10].

Prior studies have already underscored superior outcomes in patients who experience weight loss compared to those who do not [11]. In a cohort study involving 7,270 patients evaluating kidney transplant results, higher graft survival was observed in obese patients who lost more than 10% of their weight compared to obese patients who did not undergo weight loss [12]. Furthermore, weight reduction could enhance the eligibility of individuals with obesity for transplantation, potentially leading to improvements in both short-term and long-term outcomes [13, 14].

Numerous programs have incorporated robotic technology to minimize surgical risks in severely obese candidates, expanding therapeutic possibilities [15–18]. Nevertheless, the ideal timing for performing bariatric surgery in relation to kidney transplantation remains a topic of ongoing debate. This study carried out a retrospective evaluation of kidney transplant recipients with BMI >35 kg/m^2^. It aimed to provide data on those who received both sleeve gastrectomy (SG) and kidney transplantation (KT) simultaneously, as well as on patients who underwent SG and KT at different times, either before or after. In addition, the acceptance levels of the bariatric surgery among different scenarios were assessed.

## Materials and Methods

### Study Design and Patient Population

We conducted a retrospective study on patients who received kidney transplants (KT) and received bariatric surgical consultation at our center from April 2012 to August 2022. This study was approved by IRB# 2022-1122.

The multidisciplinary transplant recipient review committee at the University of Illinois Kidney Transplant Program determined the patient’s eligibility for kidney transplantation. In our cohort, patients underwent both open kidney transplant (OKT) and robotic-assisted kidney transplant (RKT). Per protocol, adult patients (aged >18 years) were considered eligible for RKT if they had a body mass index (BMI) of ≥35 kg/m^2^ at the time of listing but excluded in the presence of severe iliac atherosclerosis. Following the 1991 National Institutes of Health guidelines for bariatric procedures, all patients with a BMI exceeding 35 kg/m^2^ were recommended to undergo consultation for bariatric surgery [19]. All patients with ESRD and a BMI greater than 35 kg/m^2^ and a potential living donor were considered for a combined procedure. Patients on the waiting list for deceased organ transplants were offered the opportunity to participate in a weight loss program and undergo a consultation for bariatric surgery, considering sleeve gastrectomy (SG) before or after the transplant surgical procedure. For accuracy, non-surgical weight management options were offered in patients with a BMI lower than 35 kg/m^2^. However, as per our protocol, surgical management remains the primary option for candidates with a BMI over 35 kg/m^2^.

In our study population, we categorized individuals into four distinct groups. *Group 1* included patients who underwent kidney transplantation after sleeve gastrectomy. *Group 2* comprised recipients who underwent a simultaneous KT and SG. *Group 3* was composed of patients who received KT before SG. Additionally, we established *Group 4*, which consisted of patients who underwent a consultation for bariatric surgery but declined to proceed with the surgical procedure.

Only recipients with at least 1-year of follow-up from the date of the KT and SG were included in the analysis. Patients who had undergone a bariatric surgical procedure other than sleeve gastrectomy and recipients who underwent simultaneous kidney-pancreas transplantation were excluded from the analysis.

As a result of constraints in the electronic health records data, we limited the sub-group analysis to examine the acceptance rate of the bariatric surgical consultation only for patients between June 2018 and August 2022. Moreover, In the calculation of the acceptance rate for the combined KT and SG, it’s noteworthy that nine patients from a prior randomized clinical trial initially agreed to undergo the combined procedure but were subsequently randomized into the control group. These patients were classified as acceptors, irrespective of whether they ultimately underwent the procedure.

### Data Collection and Statistical Analysis

Pre-transplant and post-transplant characteristics were collected through electronic health records. These included recipient characteristics as age, sex, ethnicity, height, weight, BMI (Body Mass Index) at the time of the KT and SG, comorbidities, dialysis information, donation type (living or deceased), type of surgery performed for the transplantation (OKT or RKT), length of surgery, length of stay, readmission rate, glomerular filtration rate (GFR) at 6 and 12 months post-transplant, serum creatinine (SCr) at 3, 6 and 12 months post-transplant, BMI post-SG at 3, 6 and 12 months, and 1-year organ and patient survival.

Excess weight loss (%) was calculated as follows: excess weight loss (%) = [(initial excess weight – postoperative excess weight)/initial excess weight] × 100, where excess weight (kg) = initial weight – ideal weight, and ideal weight (kg) = 23 × height^2^.

Comprehensive descriptive analyses of all variables were performed. Qualitative variables were presented as counts and percentages. Normally, distributed quantitative variables were computed as mean ± standard deviation, and nonnormally distributed data were presented as median (range). Analysis was exclusively conducted among specific combinations (limitations section for more in-depth information). A *p*-values <.05 was considered statistically significant. The software used was IBM SPSS Statistics for Windows [20–22].

### Immunosuppressive Regimen

Induction therapy, alongside a methylprednisone bolus of 500 mg, was administered to all patients. The treatment for the majority included rabbit antithymocyte globulin at a dosage of 1.5 mg/kg daily from postoperative day (POD) 0–4. African American patients, ABO incompatible, or with a positive cross-match received thymoglobulin induction. Basiliximab at 20 mg on POD 0 and 4, or alemtuzumab at 30 mg on POD 0, was administered to the remaining patients. Following this, maintenance immunosuppression was provided using either tacrolimus or cyclosporine, with tacrolimus levels targeted at 7–10 ng/mL for the initial month post-transplantation, adjusting to 3–7 ng/mL afterwards. Cyclosporine levels were aimed at 200–250 ng/mL for the first month, reducing to 150–200 ng/mL subsequently. Cyclosporine, in particular, was primarily utilized for patients considered at risk for diabetes following transplantation, in combination with mycophenolic acid and a brief 5-day steroid taper. During the induction phase, antimicrobial prophylaxis was applied. For patients or donors with positive cytomegalovirus serologies, treatment with valganciclovir at 450 mg/day was prescribed for 6 months, while those without positive serologies received a one-month course of acyclovir to prevent herpes simplex virus. Desensitization, involving a mix of plasmapheresis and intravenous immunoglobulin, was necessary for patients who were ABO incompatible, cross-match positive, or had a high panel reactive antibody count.

## Results

### Cohort Characteristics

After a retrospective analysis of our database, we identified a total of four groups. *Group 1* included a total of 3 patients with living donors and 21 patients with deceased donors; *Group 2–*31 patients with living donors and 1 patient with deceased donor; *Group 3–*19 patients with living donors and 12 patients with deceased donors; *Group 4*–12 patients with living donors and 32 patients with deceased donors. Additional details can be found in Table 1; Supplementary Tables S1, S2.

**TABLE 1:** Living donation with robotic-assisted approach - *Group 2* (KT + SG), *Group 3* (KT before SG), *Group 4* (Only KT).

Characteristics	Only KT (N = 12)	KT + SG (N = 24)	KT before SG (N = 17)	*p*
Age* (years), mean ± SD	52.9 (0.5)	43.3 (10.2)	53.7 (2.4)	0.376
Male gender, n (%)	9 (75)	10 (41.7)	7 (41.2)	0.124
Ethnicity and race, n (%)				
• Caucasian	4 (33.3)	8 (33.3)	2 (11.8)	0.412
• African-American	5 (41.7)	11 (45.8)	11 (64.7)	
• Hispanic	2 (16.7)	5 (20.8)	2 (11.8)	
• Asian	0	0	0	
• Other	1 (8.3)	0	2 (11.8)	
BMI* (kg/m2), mean ± SD	43.7 (4.1)	44.1 (5.3)	40.7 (0.4)	0.964
Co-morbidities, n (%)				
• Hypertension	11 (91.7)	24 (100)	17 (100)	0.207
• Hyperlipidemia	10 (83.3)	14 (58.3)	4 (23.5)	**0.029**
• Diabetes mellitus	6 (50)	15 (62.5)	3 (17.6)	0.072
• High cardiac risk (EF < 45%)	6 (50)	9 (37.5)	2 (11.8)	0.177
Pretransplant dialysis (months), median (range)	14 (28)	11.5 (96)	1 (97)	0.119
Time frame KT – SG (years), median (range)	NA	NA	2.24 (10.7)	NA
BMI^✖^ (kg/m2), median (range)				
• 3 months	44.2 (6.2)	37.2 (24.7)	38.1 (19.2)	**0.023**
• 6 months	45 (6.4)	34.2 (25)	35.1 (20)	**< 0.001**
• 12 months	46.7 (5.2)	35.3 (26)	33.3 (20)	**< 0.001**
EWL^✖^ (%), median (range)				
• 3 months	4 (8)	26.2 (36.8)	34.6 (28.6)	0.213
• 6 months	1.6 (7.2)	31.7 (41.5)	43.1 (37.3)	0.113
• 12 months	−1.4 (3.6)	27.1 (67.1)	54.3 (61.1)	**< 0.001**

Abbreviations: EWL, excess weight loss; GFR, glomerular filtration rate; KT, kidney transplant; NA, not available. *at the time of transplantation. ^✖^delta between weight at the follow-up and weight at the time of sleeve gastrectomy (or KT, for the control group). The bold values represents the statistical significance.

Table 1 illustrates only patients in *Group 2* (KT + SG), *Group 3* (KT before SG), and *Group 4* (Only KT) with living donation with robotic-assisted approach. A total of 7 patients who underwent the open surgical approach is detailed alongside patients who underwent the robotic-assisted approach in Supplementary Table S1. Indeed, as per protocol they were not considered for the robotic approach due to presence of severe iliac atherosclerosis. Supplementary Table S2 presents cases involving deceased donation, where both the robotic-assisted and open approaches are listed across the four distinct groups.

### BMI and Excess Weight Loss

**FIGURE 1:**
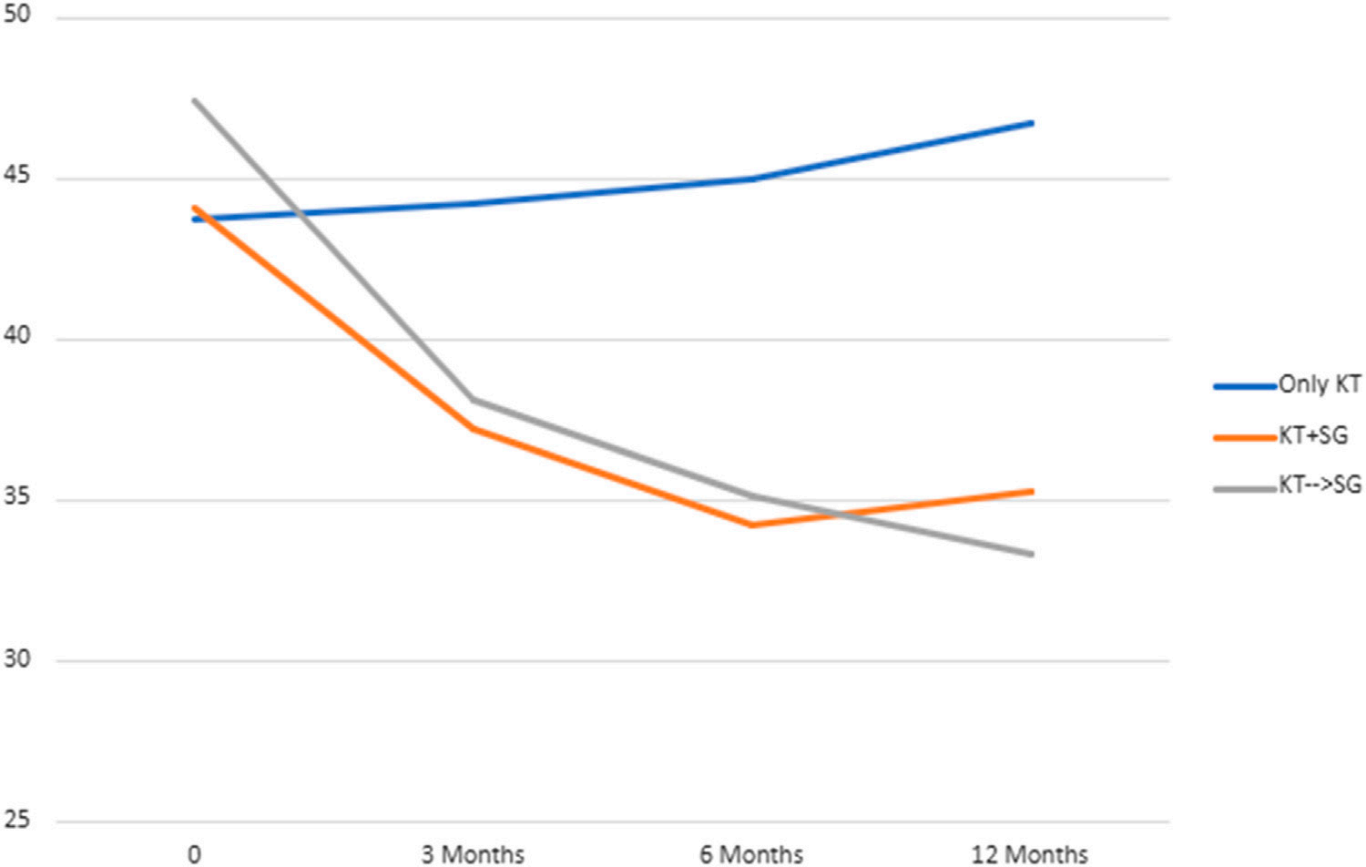
BMI trend for living donation with robotic-assisted approach - *Group 2* (KT + SG), *Group 3* (KT before SG), *Group 4* (Only KT).

**FIGURE 2:**
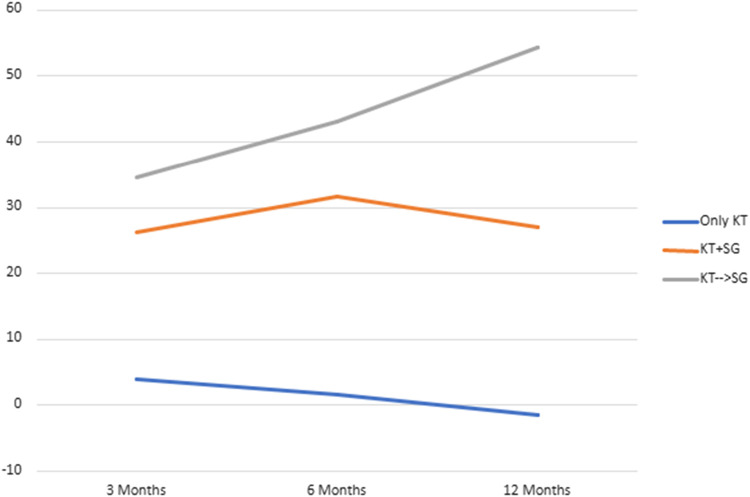
Estimated weight loss percentage trend for living donation with robotic-assisted approach - *Group 2* (KT + SG), *Group 3* (KT before SG), *Group 4* (Only KT).

BMI values and EWL percentages at different time points (3 months, 6 months, and 12 months) post-surgery were compared across the groups (*Group 2* (KT + SG), *Group 3* (KT before SG), *Group 4* (Only KT)) in Table 1. Significant differences were observed in BMI at 3 months (*p* = 0.023), 6 months (*p* < 0.001), and 12 months (*p* < 0.001). Similarly, EWL percentages differed significantly at 12 months (*p* < 0.001), with smaller variations at 3 and 6 months. These differences suggest changing body weight trends among the groups over time. Nonetheless, upon comparing only *Group 2* and *Group 3* (Table 2), no statistical significance was observed at the same time points for BMI and EWL. Figure 1 and Figure 2 illustrate respectively the BMI and the estimated weight loss percentage trends for living donation with robotic-assisted approach between Group 2 (KT + SG), Group 3 (KT before SG), Group 4 (Only KT).

**TABLE 2:** Living donation with robotic-assisted approach - Group 2 (KT + SG) and Group 3 (KT before SG).

Characteristics	KT + SG (N = 24)	KT before SG (N = 17)	*p*
Age* (years), mean ± SD	43.3 (10.2)	53.7 (2.4)	0.516
Male gender, n (%)	10 (41.7)	7 (41.2)	0.615
Ethnicity and race, n (%)			
• Caucasian	8 (33.3)	2 (11.8)	0.119
• African-American	11 (45.8)	11 (64.7)	
• Hispanic	5 (20.8)	2 (11.8)	
• Asian	0	0	
• Other	0	2 (11.8)	
BMI* (kg/m2), mean ± SD	44.1 (5.3)	40.7 (0.4)	0.782
BMI at Sleeve Gastrectomy (kg/m2), mean ± SD	44.1 (5.3)	47.4 (6.7)	0.088
Co-morbidities, n (%)			
• Hypertension	24 (100)	17 (100)	1
• Hyperlipidemia	14 (58.3)	4 (23.5)	0.109
• Diabetes mellitus	15 (62.5)	3 (17.6)	**0.022**
• High cardiac risk (EF < 45%)	9 (37.5)	2 (11.8)	0.160
Pretransplant dialysis (months), median (range)	11.5 (96)	1 (97)	0.386
BMI^✖^ (kg/m2), median (range)			
• 3 months	37.2 (24.7)	38.1 (19.2)	0.170
• 6 months	34.2 (25)	35.1 (20)	0.280
• 12 months	35.3 (26)	33.3 (20)	0.467
EWL^✖^ (%), median (range)			
• 3 months	26.2 (36.8)	34.6 (28.6)	0.318
• 6 months	31.7 (41.5)	43.1 (37.3)	0.406
• 12 months	27.1 (67.1)	54.3 (61.1)	0.925

Abbreviations: EWL, excess weight loss; GFR, glomerular filtration rate; KT, kidney transplant; NA, not available. *at the time of transplantation. ^✖^delta between weight at the follow-up and weight at the time of sleeve gastrectomy (or KT, for the control group). The bold values represents the statistical significance.

In Supplementary Table S3, the internal group statistics for *Group 1* (KT after SG) are detailed. The time frame from Sleeve Gastrectomy (SG) to Kidney Transplant (KT) is reported as 1.7 years (median range: 6.1). The mean BMI at Sleeve Gastrectomy is 43.8 kg/m2 (SD: 5.6), and a paired sample test reveals a significant BMI decrease (*p* < 0.001) at the time of Kidney Transplant, where the mean BMI is 34.8 kg/m2 (SD: 5.1). The Pearson correlation coefficient for Delta BMI at SG and the time frame SG to KT is −0.181, with a *p*-value of 0.40, suggesting no significant correlation.

Supplementary Table S4 provides the internal group statistics for *Group 3* (KT before SG). The time frame from KT to SG is reported as 2.2 years (median range: 10.9). The mean BMI at KT is 44.5 kg/m2 (SD: 6.6), and the paired sample test yields a *p*-value of 0.38, indicating no statistically significant change in BMI at the time of SG, where the mean BMI is 45.3 kg/m2 (SD: 5.6). The Pearson correlation coefficient for Delta BMI at SG and the time frame KT to SG is 0.159, with a *p*-value of 0.39, suggesting no significant correlation. Both Supplementary Tables S3, S4 include patients regardless of the type of surgical approach and the type of donor.

### Graft Function and Survival, and Patient Survival

Table 3 presents data comparing *Group 2* (KT + SG) and *Group 4* (Only KT) in living donation with a robotic-assisted approach. GFR and serum creatinine measurements are provided at 6- and 12-months post-surgery. These values did not show statistical significance between the two groups, except for GFR at 12 months (*Group 2* VS *Group 4*, 62 (SD: 14.5) VS 49.3 (SD: 4.2), *p* = 0.020). Additionally, Table 3 includes 1-year graft survival percentages, with 95.8% for *Group 2*% and 91.7% for *Group 4*, and 1-year patient survival percentages of 95.8% for *Group 2*% and 100% for *Group 4*, with no statistically significant differences observed. Supplementary Tables S1, S2 offer this information for all four groups within the context of living and deceased donation, incorporating both robotic-assisted and open approaches.

**TABLE 3:** Living donation with robotic-assisted approach - Group 2 (KT + SG) and Group 4 (Only KT).

Characteristics	Only KT (N = 12)	KT + SG (N = 24)	*p*
Age* (years), mean ± SD	52.9 (0.5)	43.3 (10.2)	0.368
Male gender, n (%)	9 (75)	10 (41.7)	0.059
Ethnicity and race, n (%)			
• Caucasian	4 (33.3)	8 (33.3)	0.551
• African-American	5 (41.7)	11 (45.8)	
• Hispanic	2 (16.7)	5 (20.8)	
• Asian	**0**	0	
• Other	1 (8.3)	0	
BMI* (kg/m2), mean ± SD	43.7 (4.1)	44.1 (5.3)	0.964
Co-morbidities, n (%)			
• Hypertension	11 (91.7)	24 (100)	0.151
• Hyperlipidemia	10 (83.3)	14 (58.3)	0.134
• Diabetes mellitus	6 (50)	15 (62.5)	0.473
• High cardiac risk (EF < 45%)	6 (50)	9 (37.5)	0.473
Pretransplant dialysis (months), median (range)	14 (28)	11.5 (96)	**0.033**
Length of surgery (minutes), mean ± SD	275 (7.1)	355.7 (125.3)	**0.034**
Length of stay (days), mean ± SD	5 (1.4)	7.4 (3.4)	0.181
Readmission rate post KT, n (%)	6 (50)	15 (62.5)	0.358
GFR (mL/min), mean ± SD			
• 6 months	43 (9.1)	61.5 (17.7)	0.135
• 12 months	49.3 (4.2)	62 (14.5)	**0.020**
SCr (mg/dL), mean ± SD			
• 6 months	1.8 (0.9)	1.2 (0.2)	0.078
• 12 months	1.5 (0.4)	1.2 (0.3)	0.251
BMI^✖^ (kg/m2), median (range)			
• 3 months	44.2 (6.2)	37.2 (24.7)	**0.004**
• 6 months	45 (6.4)	34.2 (25)	**< 0.001**
• 12 months	46.7 (5.2)	35.3 (26)	**< 0.001**
EWL^✖^ (%), median (range)			
• 3 months	4 (8)	26.2 (36.8)	0.202
• 6 months	1.6 (7.2)	31.7 (41.5)	0.107
• 12 months	−1.4 (3.6)	27.1 (67.1)	**0.003**
1-year graft survival, n (%)	11 (91.7)	23 (95.8)	0.562
1-year patient survival, n (%)	12 (100)	23 (95.8)	0.667

Abbreviations: EWL, excess weight loss; GFR, glomerular filtration rate; KT, kidney transplant; NA, not available. *at the time of transplantation. ^✖^delta between weight at the follow-up and weight at the time of sleeve gastrectomy (or KT, for the control group). The bold values represents the statistical significance.

### Acceptance Rate

**FIGURE 3:**
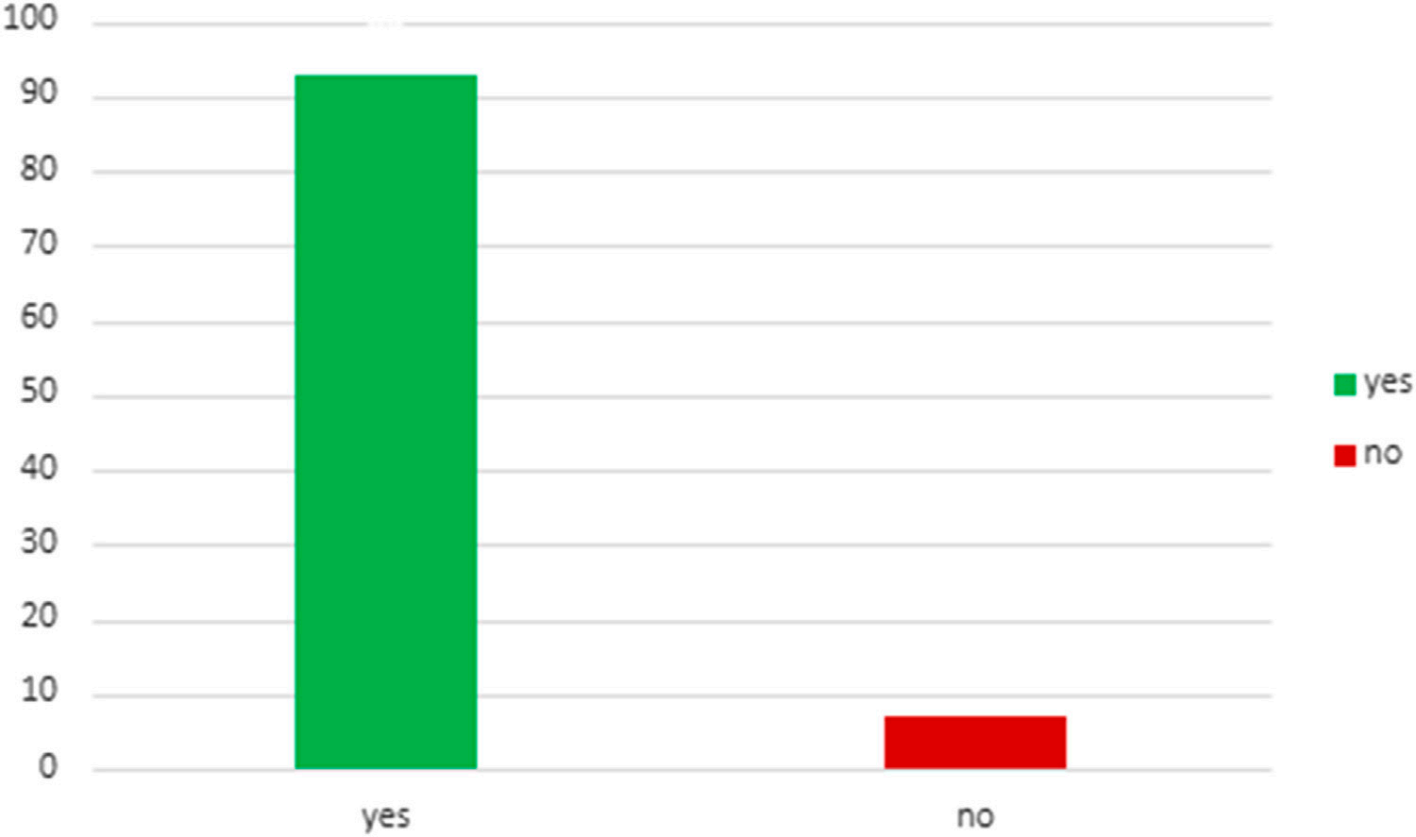
Patients with living donation who underwent KT combined with SG after consultation.

**FIGURE 4:**
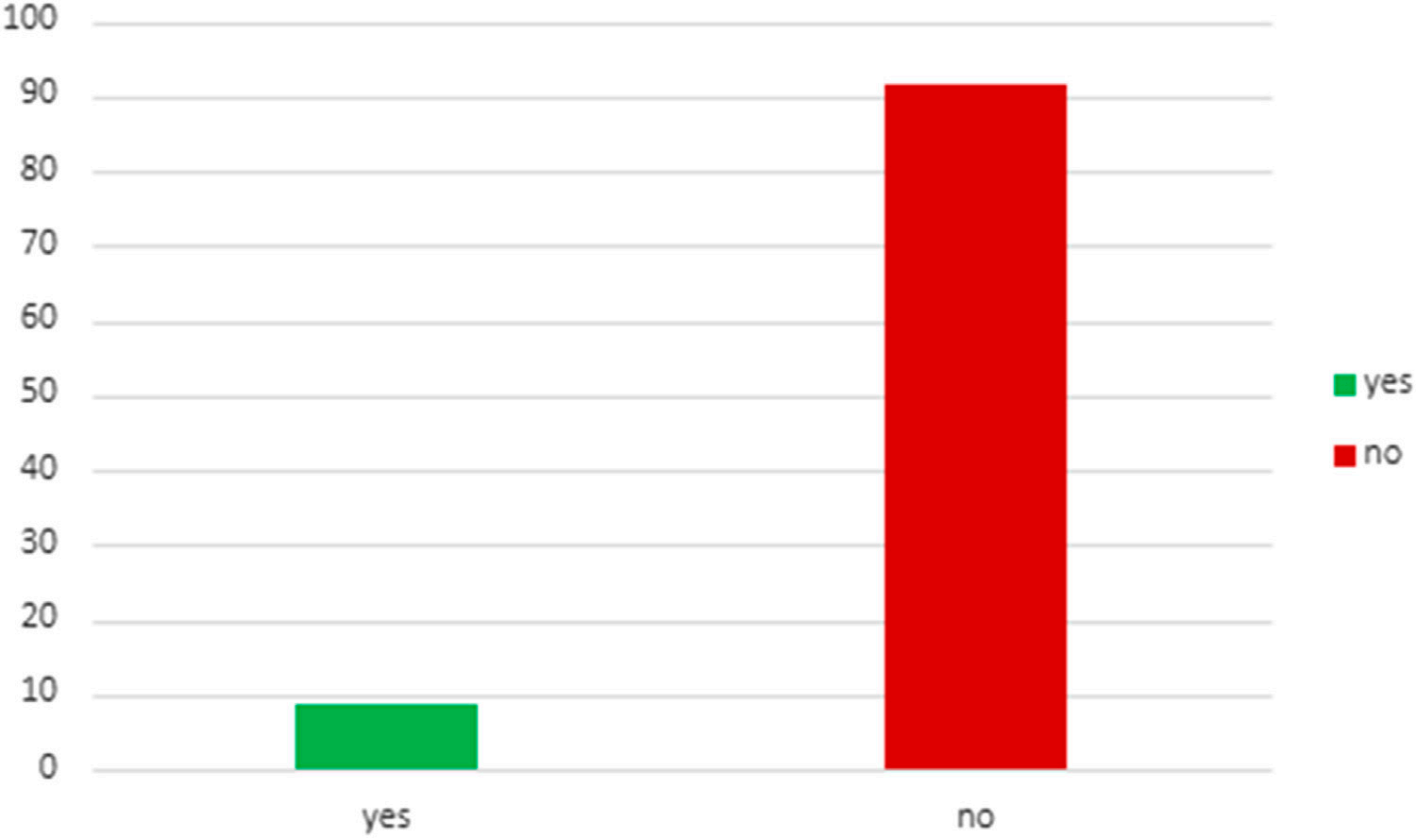
Patients with deceased donation who underwent KT with SG at any time after consultation.

Table 4 describes the acceptance rate of SG consultations and the subsequent procedures in the setting of living and deceased donor kidney transplant. Among patients with living donor, all 43 individuals accepted consultation, while 93% of them underwent kidney transplant combined with sleeve gastrectomy (SG) after consultation. For patients with deceased donor, 386 patients accepted consultation, but only 8.5% of them proceeded with a SG at some point (before or after KT). This data underscores a notable contrast (*p* < 0.001) in the acceptance of consultations and the actual performance of the SG between living and deceased donor scenarios, highlighting the higher likelihood of proceeding with the combined procedure in the former group. Figures 3, 4 illustrates these findings.

**TABLE 4:** Acceptance rate between June 2018 and August 2022.

Characteristics	N	Yes	No	%
Living Donor				
- accepted consultation^✖^	43	43	0	100
- underwent KT combined with SG after consultation*	43	40	3	93
Deceased Donor				
- accepted consultation^✖^	386	65	321	16.8
- underwent KT with SG at any time*	386	33	353	8.5

^✖^ *p* < 0.001, *p < 0.001.

## Discussion

In this retrospective study, we described four patient groups who underwent kidney transplantation and received bariatric surgical consultation at the University of Illinois at Chicago from April 2012 to August 2022. *Group 1* included patients who underwent kidney transplantation after sleeve gastrectomy, *Group 2* comprised recipients who underwent a simultaneous KT and SG, *Group 3* was composed of patients who received KT before SG, and *Group 4* consisted of patients who underwent a consultation for bariatric surgery but declined to proceed with the surgical procedure.

Obesity impacted 670 million adults worldwide in 2016. In the United States, the obesity rate has been steadily increasing since the 1980s, with a projected prevalence of 48.9% among American adults by 2030 [23, 24]. An increasing number of studies find obesity as a driver of chronic kidney disease progression, and the mechanisms are complex and include hemodynamic changes, inflammation, oxidative stress, and activation of the renin-angiotensin-aldosterone system [25].

Despite increased risk for early surgical complications and delayed graft function in patients with obesity, experience from multiple centers demonstrate a clear survival benefit of transplantation over dialysis, and comparable graft and patient survival rates to nonobese recipients. However, to date, obesity is associated with a lower rate of referral and waitlisting, and lower likelihood of kidney transplantation [26]. Between January 2009 and December 2018, we conducted a retrospective analysis of our cohort of patients undergoing RKT. This analysis comprised 239 patients, with a median BMI of 41.4 kg/m^2^. The robotic approach has led to a statistically significant decrease in surgical site infections within this population of obese recipients, while maintaining graft and patient survival rates comparable to those of the nonobese population [18]. Based of this experience, in our current clinical protocol, we abstain from employing a definitive BMI threshold for kidney recipients. Our intermediate and extended-term findings corroborate the conjecture that BMI, in isolation, is not an optimal metric for precluding transplant eligibility [27].

The optimal strategy for managing obesity in the context of ESRD patients remains uncertain. Implementing lifestyle modifications for substantial and effective weight loss poses a challenge and is frequently unsuccessful in individuals with obesity [28]. Introducing bariatric surgery before kidney transplant has become increasingly popular, with studies have shown acceptable morbidity and mortality rates [29–31]. However, a drawback to this strategy is the prolonged wait for a kidney transplant, coupled with elevated risks during dialysis [32]. Additionally, in the context of living organ donation, it’s crucial to recognize that the availability of the organ is temporary. Thus, any factors contributing to a prolonged kidney transplant process may risk the feasibility of the living donor. This emphasizes the need to streamline the transplant procedure for both its success and to preserve the readiness of the living donor. One potential resolution to these issues involves combining sleeve gastrectomy and kidney transplant in the same operative time. This approach facilitates a more rapid transplantation process, requiring only a single administration of general anesthesia. As per our current protocol, all patients with ESRD and a BMI greater than 35 kg/m^2^ and a potential living donor are considered for a combined procedure. Patients on the waiting list for deceased organ transplants are offered the opportunity to participate in a weight loss program and undergo a consultation for bariatric surgery, considering sleeve gastrectomy before or after the transplant surgical procedure.

In our previously randomized study, we demonstrated the efficacy and safety of the combined approach (11 patients with robotic sleeve gastrectomy and robotic-assisted kidney transplant VS 9 patients with robotic-assisted kidney transplant only) [33]. In this study, we examine a broader cohort within the combined group, incorporating details about two additional patient populations (KT after SG and KT before SG) and reporting the acceptance rate of bariatric surgery in our cohort.

Earlier articles have already addressed the outcomes of bariatric surgery both pre and post kidney transplantation [34, 35]. In their meta-analysis, Fernando et al. demonstrated that bariatric surgery is both safe and efficacious in patients with ESRD prior to KT and in those post KT, suggesting that SG should be strongly considered as part of the workup of the high BMI kidney recipient. In our study, we introduce a novel variable into the equation, illustrating that individuals undergoing simultaneous SG and KT exhibit comparable BMI and EWL trends to those of patients undergoing sleeve gastrectomy following kidney transplant. Consistent with earlier studies, we also observed a noteworthy reduction in mean BMI among patients undergoing Sleeve Gastrectomy (SG) before Kidney Transplant (KT) within a median of 1.7-year timeframe. Conversely, for patients who underwent SG after KT within a median of 2.2-year timeframe, there was no statistically significant change in mean BMI.

Graft and patient survivals, in robotic-assisted living kidney donation, were similar with no statistically significant differences noted between Group 2 (KT + SG) and Group 4 (Only KT). At the 12-month, the combined group exhibited a superior GFR compared to the KT alone group. Although long-term graft survival data is currently unavailable, we hypothesize that addressing obesity could play a pivotal role in enhancing extended graft survival. The observed improvement in GFR within the initial year suggests a positive trajectory for renal function in the combined approach, prompting the expectation that early management of obesity may contribute to sustained graft health over the long term. Also, our larger cohort did not exhibit a statistically significant increase in the readmission rate between the two groups, a contrast to our previous randomized study findings [33]. In that earlier study, the KT + SG group had a higher readmission rate attributed to nausea and vomiting leading to dehydration and acute kidney injury (AKI). To address this issue, we implemented a strategy involving the placement of a peripherally inserted central catheter on the day of discharge and prescribed home intravenous fluid repletion with 2 L/day of crystalloid solution for the initial postoperative month. Our recent findings indicate the success of this strategy.

Discussing the management of immunosuppression in the group undergoing combined procedures is essential. The most prevalent bariatric surgeries are sleeve gastrectomy and Roux-en-Y gastric bypass (RYGB) [36, 37]. Sleeve gastrectomy is mainly a restrictive surgery that involves the removal of a large section of the stomach, while RYGB is both restrictive and malabsorptive, requiring the creation of a small stomach pouch and a Roux-en-Y gastrojejunostomy. Differing from sleeve gastrectomy, RYGB impacts the absorption processes and is specifically known to alter the pharmacokinetic dynamics of immunosuppressive drugs [38]. This specific characteristic of sleeve gastrectomy did not present any obstacles in adhering to the standard of care immunosuppression regimens set by the University of Illinois at Chicago Kidney Transplant Program.

While bariatric surgery has proven effective in this patient cohort, it’s crucial to consider patients’ perspectives on undergoing an additional procedure alongside the transplant. Initially, we observed significant differences in acceptance rates when proposing a combined procedure for living donor recipients versus two separate procedures for deceased donor recipients. Consequently, we conducted a more thorough investigation into the consultation rate and acceptance of the procedure, revealing substantial discrepancies in results (93% vs. 8.5%). Our interpretation of this trend is that the idea of addressing two issues in a single hospitalization is appealing to patients. It effectively minimizes logistical challenges and lessens the burden on families. However, it is crucial to bear in mind that both procedures entail intricate post-surgery care, ranging from managing immunosuppressive regimens to adapting to the lifestyle changes post-bariatric surgery. Given this, a thorough psychological assessment (evaluating psychological issues/comorbidities, social support, motivation, and capacity to manage the demands post-surgeries) is essential for the success of a combined approach, where the psychological burden may be even greater than usual [39, 40]. Indeed, a weight regain 6 months post-operation in the combined group, as opposed to the sleeve gastrectomy group following kidney transplant, could stem from the demanding nature of post-transplant care, possibly overshadowing patients’ ongoing commitment to their sleeve gastrectomy education. While a more in-depth qualitative study is essential for a comprehensive understanding of this trend, the practicality of achieving comparable clinical outcomes with combined kidney transplant and sleeve gastrectomy proves beneficial in addressing both ESRD and obesity, thereby expanding the reach to more patients.

While a similar study comparing bariatric surgery before, combined, and after liver transplant has been previously published, our paper is, to the best of our knowledge, the first to present data for these groups in the context of kidney transplant and to explore acceptance rates for bariatric surgery [41].

### Limitations

Our study holds considerable strength as the inaugural exploration of acceptance rates and timing for sleeve gastrectomy in kidney transplant recipients. However, our study does have certain limitations. Firstly, it is essential to acknowledge the inherent limitations associated with its retrospective design. Secondly, the comparative analysis across groups posed challenges due different type of donor (living VS deceased) and surgical approach (open VS robotic), consequently, *p*-values were selectively considered in specified contexts. Moreover, the creatinine-based GFR might be affected in patients experiencing substantial muscle mass loss.

Despite these constraints, our study serves as a foundational step in understanding the complex dynamics related to the surgical management of obesity in this specific patient population, paving the way for future prospective investigations to further elucidate these considerations.

### Conclusion

In summary, our retrospective investigation indicates that the simultaneous kidney transplant and sleeve gastrectomy resulted in successful weight loss compared to kidney transplant alone, while maintaining similar rates of graft and patient survival. We observed a consistent trend in 1-year BMI and excess weight loss among patients who underwent simultaneous SG and KT compared to those who had KT before SG. Additionally, in a median time frame of 1.7 years, patients who underwent SG prior to KT showed a statistically significant reduction in BMI at the time of the transplant. Notably, our study highlights that patients offered the combined procedure were significantly more likely to undergo surgery compared to those for whom sleeve gastrectomy was presented at a different operative time than the transplant. Further prospective studies are necessary to obtain additional insights from the combined group.

## Data Availability

The data analyzed in this study is subject to the following licenses/restrictions: None. Requests to access these datasets should be directed to alessandro.martinino@uic.edu.

## Abbreviations

BMI, body mass index; CKD, chronic kidney disease; ESRD, end-stage renal disease; EWL, excess weight loss; HbA1c, glycated hemoglobin; KT, kidney transplantation alone; OKT, open kidney transplant; RKT, robotic kidney transplant; SG, sleeve gastrectomy.
