# Supplementary material for: Timing Considerations for Sleeve Gastrectomy in Kidney Transplant Patients: A Single Center Evaluation
Source: Transpl Int. 2024 Jun 18;37:12690. doi: 10.3389/ti.2024.12690 (PMC11217181; doi:10.3389/ti.2024.12690)
Supplement: Supplementary file 1 [file DataSheet1.docx]

**Table S1**. Living donation with robotic-assisted approach + open approach - *Group 1* (KT after SG), *Group 2* (KT+SG), *Group 3* (KT before SG), *Group 4* (Only KT).

| **Characteristics** | **KT after SG**  **(N=3)** | **KT + SG**  **(N=31)** | **KT before SG**  **(N=19)** | **Only KT**  **(N=12)** |
| --- | --- | --- | --- | --- |
| Age* (years), mean ± SD | 42 (2.6) | 43.7 (12.5) | 44.2 (9.9) | 52.9 (0.5) |
| Male gender, n (%) | 2 (66.7) | 14 (45.2) | 7 (36.8) | 9 (75) |
| Ethnicity and race, n (%)   - Caucasian - African-American - Hispanic - Asian - Other | 1 (33.3)  2 (66.7)  0  0  0 | 13 (41.9)  12 (38.7)  5 (16.1)  0  1 (3.2) | 2 (10.5)  12 (63.2)  3 (15.8)  0  2 (10.5) | 4 (33.3)  5 (41.7)  2 (16.7)  0  1 (8.3) |
| BMI* (kg/m2), mean ± SD | 32.8 (8.6) | 44 (5.6) | 44.1 (7.2) | 43.7 (4.1) |
| BMI at Sleeve Gastrectomy (kg/m2), mean ± SD | 46.2 (5.9) | 43.4 (5.1) | 46.1 (5.5) | NA |
| Co-morbidities, n (%)   - Hypertension - Hyperlipidemia - Diabetes mellitus - High cardiac risk (EF < 45%) | 3 (100)  1 (33.3)  1 (33.3)  1 (33.3) | 31 (100)  19 (61.3)  20 (64.5)  13 (41.9) | 19 (100)  9 (47.4)  4 (21.1)  8 (42.2) | 11 (91.7)  10 (83.3)  6 (50)  6 (50) |
| Pretransplant dialysis (months), median (range) | 34 (7) | 10 (96) | 14 (97) | 14 (28) |
| Time frame KT – SG (years), median (range) | 0.6 (1.2) | NA | 2 (2.6) | NA |
| Length of stay (days), mean ± SD | 5 (2.6) | 7.2 (3.5) | 6.1 (3) | 5 (1.4) |
| Readmission rate post KT, n (%) | 1 (33.3) | 20 (64.5) | 6 (31.6) | 6 (50) |
| GFR (ml/min), mean ± SD   - 6 months - 12 months | 70.3 (6.3)  86.9 (4.5) | 60 (17.5)  60.7 (14.5) | 57.5 (19.4)  60 (15.7) | 43 (9.1)  49.3 (4.2) |
| SCr (mg/dl), mean ± SD   - 6 months - 12 months | 1.2 (0.1)  1.1 (0.1) | 1.3 (0.3)  1.3 (0.3) | 1.4 (0.4)  1.3 (0.4) | 1.8 (0.9)  1.5 (0.4) |
| BMI^✖^ (kg/m2), median (range)   - 3 months - 6 months - 12 months | 32.9 (21)  31.4 (19.1)  30 (16.1) | 38 (25.1)  35.4 (27.6)  37.4 (26) | 38.1 (23)  35.1 (25.3)  33.3 (33.6) | 44.2 (6.2)  45 (6.4)  46.7 (5.2) |
| EWL^✖^ (%), median (range)   - 3 months - 6 months - 12 months | 25.7 (14.9)  42.3 (52.3)  60 (37.9) | 26.6 (44.3)  31.1 (60.1)  25.6 (67.2) | 29.6 (47.2)  38 (60.4)  54.3 (87) | 4 (8)  1.6 (7.2)  -1.4 (3.6) |
| 1-year graft survival, n (%) | 3 (100) | 29 (93.5) | 19 (100) | 11 (91.7) |
| 1-year patient survival, n (%) | 3 (100) | 29 (93.5) | 19 (100) | 12 (100) |

Abbreviations: EWL, excess weight loss; GFR, glomerular filtration rate; KT, kidney transplant; NA, not available.
*at the time of transplantation.
^✖^delta between weight at the follow-up and weight at the time of sleeve gastrectomy (or KT for the control group).

**Table S2**. Deceased donation with robotic-assisted approach + open approach – *Group 1* (KT after SG), *Group 2* (KT+SG), *Group 3* (KT before SG), *Group 4* (Only KT).

| **Characteristics** | **KT after SG**  **(N=21)** | **KT + SG**  **(N=1)** | **KT before SG**  **(N=12)** | **Only KT**  **(N=32)** |
| --- | --- | --- | --- | --- |
| Age* (years), mean ± SD | 58.7 (9.6) | 68.1 | 45.2 (7.8) | 50.4 (12.7) |
| Male gender, n (%) | 13 (61.9) | 1 (100) | 6 (50) | 17 (53.1) |
| Ethnicity and race, n (%)   - Caucasian - African-American - Hispanic - Asian - Other | 5 (23.8)  13 (61.9)  3 (14.3)  0  0 | 0  1 (100)  0  0  0 | 2 (16.7)  8 (66.7)  2 (16.7)  0  0 | 6 (18.8)  23 (71.9)  3 (9.4)  0  0 |
| BMI* (kg/m2), mean ± SD | 34.6 (4.3) | 51.5 | 45.3 (5.6) | 42.9 (5.8) |
| BMI at Sleeve Gastrectomy (kg/m2), mean ± SD | 44.1 (3.4) | 51.5 | 44.1 (5.7) | NA |
| Co-morbidities, n (%)   - Hypertension - Hyperlipidemia - Diabetes mellitus - High cardiac risk (EF < 45%) | 21 (100)  8 (38.1)  13 (61.9)  7 (33.3) | 1 (100)  1 (100)  1 (100)  1 (100) | 12 (100)  5 (41.7)  5 (41.7)  5 (41.7) | 29 (90.6)  7 (21.9)  19 (59.4)  11 (34.4) |
| Pretransplant dialysis (months), median (range) | 86 (172) | 108 | 87 (123) | 74 (175) |
| Time frame KT – SG (years), median (range) | 1.8 (6.1) | NA | 2 (6.3) | NA |
| Length of stay (days), mean ± SD | 7.9 (2.4) | 14 | 7.7 (4.2) | 10 (12.7) |
| Readmission rate post KT, n (%) | 7 (33.3) | 1 (100) | 4 (33.3) | 17 (53.1) |
| GFR (ml/min), mean ± SD   - 6 months - 12 months | 50.6 (14)  46.9 (25.5) | 27.5  25.9 | 50.5 (9.8)  56.9 (55.7) | 51.4 (21.2)  47.8 (21.7) |
| SCr (mg/dl), mean ± SD   - 6 months - 12 months | 1.6 (0.4)  1.6 (0.6) | 2.2  2.2 | 1.5 (0.2)  1.3 (0.1) | 1.7 (0.7)  1.9 (0.9) |
| BMI^✖^ (kg/m2), median (range)   - 3 months - 6 months - 12 months | 36.2 (7.1)  34.1 (7.6)  33.3 (9.7) | 44.2  40.3  35.7 | 38.1 (15)  37.3 (16.5)  37.4 (17.7) | 42.6 (24.3)  42.8 (21.5)  41.8 (26.9) |
| EWL^✖^ (%), median (range)   - 3 months - 6 months - 12 months | 30.2 (45.6)  43.2 (65.1)  47.3 (73.4) | 32  48.6  68.9 | 13.4 (16.1)  16.9 (15.8)  16.1 (27.5) | 9.1 (73.7)  2.9 (65.5)  -1 (90.7) |
| 1-year graft survival, n (%) | 20 (95.2) | 1 (100) | 12 (100) | 30 (93.8) |
| 1-year patient survival, n (%) | 20 (95.2) | 1 (100) | 12 (100) | 30 (93.8) |

Abbreviations: EWL, excess weight loss; GFR, glomerular filtration rate; KT, kidney transplant; NA, not available.
*at the time of transplantation.
^✖^delta between weight at the follow-up and weight at the time of sleeve gastrectomy (or KT for the control group).

**Table S3**. *Group 1* (KT after SG) – Inside group statistics.

| N | 24 |  |
| --- | --- | --- |
| Time frame SG – KT (years), median (range) | 1.7 (6.1) |  |
| BMI at Sleeve Gastrectomy (kg/m2), mean ± SD | 43.8 (5.6) | Paired Sample Test **p < 0.001** |
| BMI at Kidney Transplant (kg/m2), mean ± SD | 34.8 (5.1) |  |
| Pearson Correlation Delta BMI at SG – Time frame SG – KT | -0.181 | P=0.40 |

**Table S4**. *Group 3* (KT before SG) – Inside group statistics.

| N | 31 |  |
| --- | --- | --- |
| Time frame KT – SG (years), median (range) | 2.2 (10.9) |  |
| BMI at Kidney Transplant (kg/m2), mean ± SD | 44.5 (6.6) | Paired Sample Test p = 0.38 |
| BMI at Sleeve Gastrectomy (kg/m2), mean ± SD | 45.3 (5.6) |  |
| Pearson Correlation Delta BMI at SG – Time frame KT – SG | 0.159 | p = 0.39 |
